# Characteristics of inflammatory phenotypes among patients with asthma: relationships of blood count parameters with sputum cellular phenotypes

**DOI:** 10.1186/s13223-021-00548-z

**Published:** 2021-05-11

**Authors:** Bingqing Shi, Wei Li, Yuqiu Hao, Hongna Dong, Wenjing Cao, Jie Guo, Peng Gao

**Affiliations:** 1 Department of Respiratory Medicine, The Second Hospital of Jilin University, Changchun, 130041 Jilin China; 2 Department of Science and Education, Changchun Central Hospital, Changchun, Jilin China; 3 Department of Radiation Oncology, The Second Hospital of Jilin University, Changchun, Jilin China

**Keywords:** Asthma, Eosinophils, Induced sputum, Inflammation phenotypes, Neutrophils

## Abstract

**Background:**

There is a need to identify the asthma inflammatory phenotypes of patients to facilitate personalized asthma treatment. Sputum induction is time-consuming and requires expert clinical technique. This study aimed to assess the distribution and characteristics of asthma inflammatory phenotypes in Jilin Province, China; it also aimed to identify an easier method for characterization of an asthma phenotype, rather than sputum cellular analysis.

**Methods:**

In this study, 232 asthma patients underwent sputum induction following clinical assessment and blood collection. Inflammatory cell counts in sputum were used to classify asthma inflammatory phenotypes. Receiver operating characteristic curve and Spearman correlation coefficient analyses were used to identify correlations between clinical parameters.

**Results:**

Among the included patients, there had 52.1% paucigranulocytic, 38.4% eosinophilic, 4.3% neutrophilic, and 5.2% mixed granulocytic asthma phenotypes, respectively. In total, 129 (55.6%) patients had asthma-chronic obstructive pulmonary disease (COPD) overlap (ACO); these patients had higher proportion of smokers, higher sputum neutrophil count, worse lung function, and worse asthma control, compared with patients who had asthma alone (p < 0.05). Sputum eosinophil/neutrophil counts were positively correlated with blood eosinophil/neutrophil counts (p < 0.01). To identify the presence of sputum eosinophil proportion ≥ 3%, optimal cut-off values for blood eosinophil count and fractional exhaled nitric oxide (FeNO) were 0.2 × 10^9^/L and 30.25 ppd (area under the curve (AUC) = 0.744; AUC = 0.653, p < 0.001). AUCs did not significantly differ between FeNO and blood eosinophil count (p = 0.162), but both exhibited poor specificity (57% and 49%, respectively). To identify the presence of sputum neutrophil proportion ≥ 61%, the optimal cut-off value for blood neutrophil proportion was 69.3% (AUC = 0.691, p = 0.0003); however, this exhibited poor sensitivity (50%).

**Conclusions:**

Paucigranulocytic asthma was the most common phenotype, followed by eosinophilic asthma. Higher proportion of smokers, poor patient compliance, insufficient treatment, and poor asthma control may have been the main causes of high ACO proportion among patients in this study. Blood eosinophil/neutrophil counts exhibited poor specificity and sensitivity for prediction of airway eosinophilic/neutrophilic inflammation.

## Background

Asthma is a complex and heterogeneous disease [1] that affects approximately 358.2 million people worldwide, thus causing a substantial public health problem [2]. The Global Initiative for Asthma (GINA) has highlighted corticosteroids and biological therapies (e.g., anti-IgE antibody, anti-interleukin-5/5Rα, and anti-interleukin-4Rα) in their guidelines for asthma treatment [3]; however, these treatments primarily target Th2-mediated airway inflammation. Notably, airway inflammation in some patients with asthma exhibits neutrophil dominance, non-Th2-mediated airway inflammation, and poor response to current therapies [4, 5]. Therefore, individualized treatment regimens should be administered to patients with asthma, based on their specific asthma phenotypes [6]. Sputum induction for inflammatory cell analysis is the currently accepted method for identifying asthma inflammatory phenotype [7]. However, sputum induction requires expert clinical technique and good patient cooperation; moreover, the process is time-consuming. Some studies have proposed the possibility of fractional exhaled nitric oxide (FeNO) levels and whole blood eosinophil counts for prediction of airway eosinophilic inflammation [8, 9], but there remains insufficient evidence to support the reliability of this method, especially in China.

This study assessed the distribution and characteristic of asthma inflammatory phenotypes in Jilin Province, China, through analysis of inflammatory cells in sputum; it also identified factors related to sputum eosinophilia/neutrophilia in patients with asthma. The results will aid in research regarding alternative methods for sputum cell analysis to predict airway eosinophilic/neutrophilic inflammation and provide guidance for the individualized clinical treatment of patients with asthma.

## Methods

### Study design and patients

This cross-sectional study included eligible patients with asthma in the Department of Respiratory Medicine of Jilin University Second Hospital between June 2016 and December 2019. Each recruited patient underwent examinations of lung function, sputum induction, FeNO measurement, and routine blood analyses. Each patient completed the following questionnaires: 6-item Asthma Control Questionnaire (ACQ6), Asthma Control Test Questionnaire (ACT), Asthma Quality of Life Questionnaire (AQLQ), and Hospital Anxiety and Depression scale (HADS). The study protocol was approved by the ethics committee of the Second Hospital of Jilin University (2016-34), and all patients provided informed consent to participate.

Patient selection criteria were as follows: age ≥ 18 years; diagnosis of asthma in accordance with 2012 GINA criteria [10]; stable condition without acute exacerbation of asthma.

The main exclusion criteria for patients and healthy controls were pregnancy, cognitive impairment, malignant tumors and chronic diseases of various systems and organs, severe irreversible organ failure, chronic obstructive pulmonary disease (COPD) (except for asthma and COPD overlap [ACO]), or pulmonary diseases (except for Asthma).

Severe asthma, as defined in the 2019 GINA guidelines [3], refers to uncontrolled asthma despite adherence to maximal optimized therapy, or asthma that worsens when high-dose treatment is reduced. ACO in this study was defined in accordance with the 2015 GINA guidelines [11]: (1) a current diagnosis of asthma; (2) persistent airflow limitation (respiratory symptoms and post-BD FEV1/FVC < 0.7); (3) patient age ≥ 40 years; and/or (4) current smoker or ex-smoker status. Atopy was defined as the presence of at least one common aeroallergen or serum specific IgE against one common aeroallergen (e.g., cat, dog, house dust mites, grass pollen, tree pollen, and a mixture of molds). Low-dose inhaled corticosteroids (ICS) was defined as 200–400 ug/day budesonide (or equivalent); medium dose was defined as 400–800 ug/day budesonide (or equivalent); and high dose was defined as over 800 ug/day budesonide (or equivalent) [12]. Ex-smoker status was defined as the cessation of smoking for more than 1 year prior to the study.

### Sputum collection

Induced sputum was collected and processed using validated methods [13, 14]. Briefly, all patients underwent sputum induction with 4.5% hypertonic saline for 15 min. Sputum was treated using dithiothreitol; the total cell count of leukocytes was evaluated, single-cell smears were prepared, and inflammatory cell counts (i.e., those of eosinophils, neutrophils, macrophages, epithelial cells, and lymphocytes) were recorded.

### Asthma phenotype classification

Based on published criteria [15], a cutoff of 3% eosinophils was used to determine the presence of eosinophilic inflammation [16]; a cutoff of 61% neutrophils was used to determine the presence of neutrophilic inflammation [17]. Asthma was divided into four types: eosinophilic (sputum eosinophils ≥ 3%); neutrophilic (neutrophils ≥ 61%); mixed granulocytic (eosinophils ≥ 3% and neutrophils ≥ 61%); and paucigranulocytic (eosinophils < 3% and neutrophils < 61%).

### Statistical analysis

The study used IBM SPSS Statistics, version 25.0, for statistical analysis. The normally distributed data were expressed as mean ± SD; skewed data were expressed as median (interquartile range). Subgroups were compared using analysis of variance with a least significant difference test or a Kruskal–Wallis test, accompanied by Bonferroni correction. Spearman correlation coefficients were used to identify correlations between clinical characteristics. Categorical variables were presented as numbers (proportions) of observations, and were analyzed using the Chi-squared test. Receiver operating characteristic (ROC) curves were used to determine the optimal blood cell count values that best identified sputum neutrophilic/eosinophilic inflammation. Differences with p < 0.05 were considered statistically significant.

Forward logistic regression analysis was used to determine whether multiple covariates (forced expiratory volume in 1 s [FEV1] % predicted, FEV1/forced vital capacity [FVC], inhaled corticosteroid [ICS] therapy) could predict sputum neutrophilic/eosinophilic inflammation.

## Results

### Demographic data, functional, and inflammatory characteristics of all patients

In total, 232 patients with asthma were enrolled and successfully underwent sputum induction in this study (Table 1). Approximately 47% of the patients were men; 38.4% exhibited severe asthma. Their average age at diagnosis was 45 ± 15 years. There were 50 (21.6%) ex-smokers and 49 (21.1%) current smokers; the median (interquartile range) smoking pack-years were 16 (5.2, 16.0) and 22 (13.0, 37.3), respectively. There were 117 patients with asthma who underwent serum-specific IgE tests, of which 37 (31.6%) exhibited atopy. There were 129 (55.6%) patients with ACO. In 30.2% of patients, symptoms were well controlled (the ACT score between 20–25 points); in 25.9% of patients, symptoms were partially controlled. Additionally, 86.2% of patients had obvious triggering factors before the onset of asthma. Of all causes, upper respiratory tract infection (60.7%) was the most common. Approximately 48.3% of patients regularly received ICS treatment in the past year. Other detailed clinical characteristics are shown in Table 1. Among all patients (Table 2), 121 (52.1%) exhibited paucigranulocytic asthma (PGA), 89 (38.4%) exhibited eosinophilic asthma (EA), 12 (5.2%) exhibited mixed granulocytic asthma (MGA), and 10 (4.3%) exhibited neutrophilic asthma (NA). Compared with patients who did not receive ICS treatment, the proportion of patients with NA (among those who regularly received ICS treatment) gradually increased in accordance with the concentration of ICS (Table 3).

**Table 1:** Demographic, clinical, and inflammatory characteristics of patients with asthma

Characteristic	
N	232
Age, years	
≤ 20	8 (3.4%)
21–40	39 (16.8%)
41–60	129 (55.6%)
61–80	56 (24.1%)
Sex (M/F)	109/123
Height, cm	166 ± 7.9
Weight, kg	65 ± 11.2
Never smoker, N (%)	133 (57.3%)
Ex-smokers (n) (pack-years)	50 (16 (5.2, 16.0))
Current smokers (n) (pack-years)	49 (22 (13.0, 37.3))
Diagnosis age (y)	45 ± 15
Total controlled, N (%)	70 (30.2%)
Partial controlled, N (%)	60 (25.9%)
Severe asthma, N (%)	89 (38.4%)
Atopy (Y/N)	37/80
ACO, N (%)	118 (50.8%)
Triggering factors (Y/N)	200/32
Seasons, N (%)	114 (49.1%)
Exercise, N (%)	101 (43.5%)
URTI, N (%)	141 (60.7%)
Work, N (%)	20 (8.6%)
Gastroesophageal reflux, N (%)	14 (6.0%)
Pets, N (%)	11 (4.7%)
Food, N (%)	20 (8.6%)
Aspirin, N (%)	6 (2.6%)
Fumes, N (%)	50 (21.6%)
Rhinitis, N (%)	78 (33.6%)
Post-FEV1/FVC, %	67.9 ± 13.0
Post-FEV1% predicted	84.8 (63.9, 97.6)
FeNO (ppd)	40.9 (23.1, 77.8)
Application of ICS/(ICS + LABA) in the past year (Y/N)	112/120
≤ 3 month, N (%)	39 (16.8%)
3–6 month, N (%)	21 (9.1%)
> 6 month, N (%)	52 (22.4%)

Data are presented as mean ± SD or median (IQR), unless otherwise indicated

*ACO* Asthma-COPD overlap, *URTI* upper respiratory tract infection, *FeNO* fractional exhaled nitric oxide, *TCC* total cell count, *IQR* interquartile range

**Table 2:**
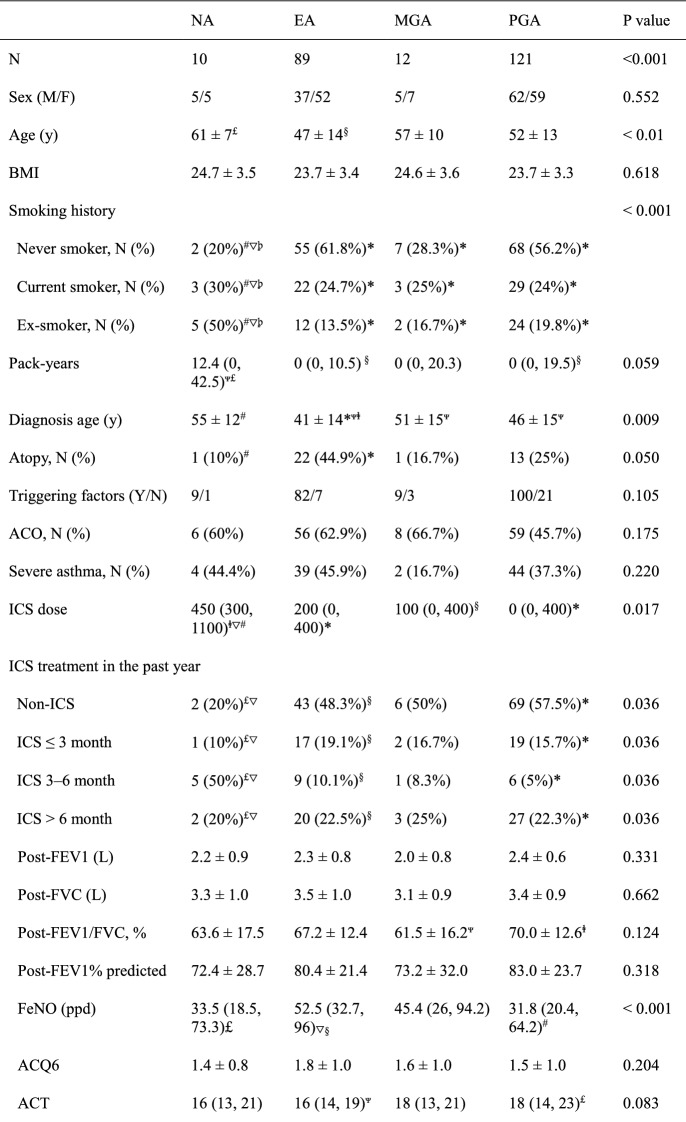

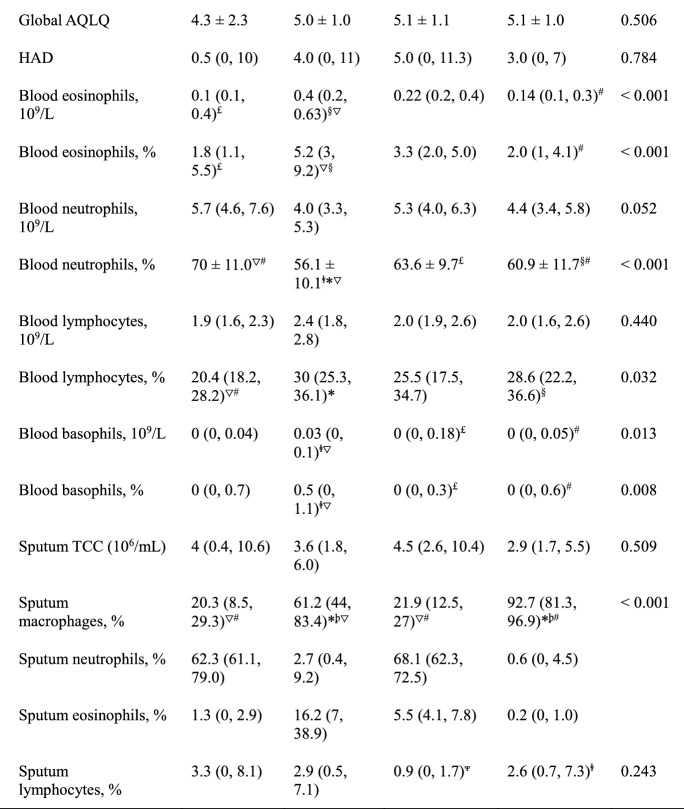
Clinical characteristics of patients with asthma according to inflammatory phenotype

Data are presented as mean ± SD or median (IQR), unless otherwise indicated

*ACO* asthma-COPD overlap, *NA* neutrophilic asthma, *EA* eosinophilic asthma, *MGA* mixed granulocytic asthma, *PG* paucigranulocytic asthma, *IQR* interquartile range

p < 0.01: *vs NA, ^#^vs EA, ^∇^vs PGA,  . p < 0.05: ^§^vs NA, ^Ψ^vs PGA, ^£^vs EA,

**Table 3:** Analysis of asthma inflammatory phenotype distribution, stratified according to inhaled corticosteroid dose category

	Steroid naïve	Low ICS dose	Middle ICS dose	High ICS dose*
NA, N (%)	2 (1.7%)	3 (4.1%)	3 (10.3%)	2 (22.2%)
EA, N (%)	43 (35.8%)	33 (44.6%)	11 (37.9%)	2 (22.2%)
MGA, N (%)	6 (5%)	5 (6.8%)	0 (0%)	1 (11.1%)
PGA, N (%)	69 (57.5%)	33 (44.6%)	15 (51.7%)	4 (44.4%)

*ICS* inhaled corticosteroid, *NA* neutrophilic asthma, *EA* eosinophilic asthma, *MGA* mixed granulocytic asthma, *PGA* paucigranulocytic asthma

p < 0.05: *vs Steroid naïve

### Demographic, functional, and inflammatory characteristics according to inflammatory phenotype

The male/female ratio did not significantly differ among the four asthma inflammatory phenotypes (p > 0.05). Notably, the average age and average age at diagnosis were highest in patients with NA, followed by those with MGA (p < 0.01) (Table 2). There were 3 (30%) current smokers and 5 (50%) ex-smokers in the NA group, and the proportion was significantly higher than in the other three phenotype groups (p < 0.001). In addition, the smoking pack-years of NA group was 12.4 (0, 42.5), which was significantly higher than that of EA and PGA groups (p < 0.05). There were no significant differences in the proportion of patients with ACO among the four groups (p > 0.05). Patients with severe asthma constituted approximately 45.9% and 44.4% of patients with EA and NA, respectively. There was 1 (10%) atopic asthma in the NA group, which was significantly different from patients with EA (22, 44.9%) (p < 0.05). Furthermore, patients with NA had the highest ICS treatment dose, whereas patients with PGA had the lowest ICS dose (p < 0.05). Compared with patients with EA, the proportion of patients not treated with ICS was significantly lower in patients with NA, while the proportion of patients who regularly used ICS for 3–6 months was significantly higher among patients with NA (p < 0.05) (Table 2).

ACQ6, AQLQ, and HADS scores were similar among the four asthma inflammatory phenotypes (Table 2). The average post-FEV_1_/FVC value of PGA group was significantly higher than that of MGA group (p = 0.046). Patients with PGA tended to have the best lung function (post-FEV_1_/FVC and post-FEV_1_% predicted), but the difference between the groups was not statistically significant. However, patients with EA had higher FeNO levels, compared with patients with PGA and those with NA (p < 0.001); they had lower ACT scores, compared with patients with PGA (p < 0.05). Patients with EA had the greatest numbers of blood eosinophils and blood lymphocytes, compared with patients with NA and those with PGA (p < 0.001). Furthermore, patients with EA had greater numbers of blood basophils, compared with patients with MGA and those with PGA (p < 0.05). Compared with patients with NA and those with MGA, patients with EA had greater numbers of sputum basophils, but this number was significantly lower than in patients with PGA (p < 0.001). Moreover, the proportions of blood neutrophils were significantly higher in patients with NA and those with MGA than in patients with EA or PGA (p < 0.001); however, the increase in blood neutrophil count was not statistically significant (p = 0.052) (Table 2).

### Clinical characteristics of patients with asthma and those with ACO

The average and diagnosed ages of patients in the ACO group were significantly greater than those in the asthma group (p < 0.001) (Table 4). Patients with ACO had a higher proportion of smokers and a higher value of smoking pack-years (p < 0.001). There were no significant differences in the asthma inflammatory phenotype distribution or the proportion of patients with severe asthma between the two groups (p > 0.05). Patients with ACO had been treated with a higher dose of ICS in the past year (p < 0.05), compared with patients who had asthma alone. A smaller proportion of patients with ACO exhibited atopy (27.7% vs 35.8%), but this difference was not statistically significant (p > 0.05). Among patients with ACO, the ACT score was lower, while the ACQ6 score was higher, compared with the asthma group (p < 0.0.05). There were no significant differences in blood eosinophil and neutrophil counts between the two groups (p > 0.05). However, patients with ACO had a higher blood monocyte count, compared with the asthma group (p < 0.05). Moreover, patients with ACO had a higher sputum total cell count (TCC) and sputum neutrophil count, compared with patients who had asthma alone (p < 0.05). There were no significant differences in sputum eosinophil and lymphocyte counts between the two groups (p > 0.05) (Table 4).

**Table 4:** Clinical characteristics of patients between asthma and asthma-COPD overlap

	Asthma	ACO	p value
N	103	129	
Sex (M/F)	52/65	56/58	0.476
Age (y)	42 ± 14	56 ± 8	< 0.001
BMI	24.1 ± 3.7	23.7 ± 3.0	0.664
NA, N (%)	4 (40%)	6 (60%)	0.514
EA, N (%)	45 (50.6%)	44 (49.4%)	0.514
MGA, N (%)	4 (33.3%)	8 (66.7%)	0.514
PGA, N (%)	64 (53.3%)	56 (46.7%)	0.514
Smoking history			< 0.001
Never smoker, N (%)	80 (68.4%)	53 (46.5%)	
Current smoker, N (%)	13 (11.1%)	36 (31.6%)	
Ex-smoker, N (%)	24 (20.5%)	25 (21.9%)	
Pack-years	0 (0, 3.3)	5.2 (0, 25.0)	< 0.001
Diagnosis age (y)	38 ± 13	50 ± 12	< 0.001
Asthma diagnosed more than 1 year, N (%)	59 (53.6%)	75 (69.4%)	0.016
Severe asthma, N (%)	48 (42.1%)	41 (37.6%)	0.494
Atopy (Y/N)	19/34	18/47	0.342
ICS dose	0 (0, 400)	250 (0, 400)	0.032
Post-FEV1/FVC, %	77.7 ± 8.8	58.0 ± 8.9	< 0.001
Post-FEV1% predicted	93.5 ± 19.9	69.7 ± 21.2	< 0.001
FeNO (ppd)	42.5 (23.3, 88.9)	39.2 (22.9, 63.4)	0.249
ACQ6	1.4 (0.5, 2.1)	1.5 (0.7, 2.2)	0.020
ACT	18.0 (14.0, 22.0)	16.0 (13.0, 20.0)	0.010
Global AQLQ	5.2 (4.2, 6.1)	5.0 (4.2, 5.8)	0.081
HAD	3.5 (0.0, 10.0)	2.0 (0.0, 10.0)	0.476
Blood eosinophils, 10^9^/L	0.3 (0.1, 0.5)	0.2 (0.1, 0.6)	0.792
Blood neutrophils, 10^9^/L	4.6 (3.9, 5.8)	3.7 (3.2, 5.0)	0.429
Blood lymphocytes, 10^9^/L	2.0 (1.5, 2.5)	2.1 (1.8, 2.8)	0.089
Blood monocytes, 10^9^/L	0.5 (0.4, 0.6)	0.5 (0.4, 0.7)	0.021
Sputum TCC (10^6^/mL)	2.7 (1.0, 5.0)	3.0 (1.9, 5.7)	0.031
Sputum macrophages, %	86.0 (59.7, 96.5)	82.0 (49.9, 93.7)	0.092
Sputum neutrophils, %	0.75 (0.0, 5.2)	1.6 (0.0, 11.5)	0.024
Sputum eosinophils, %	1.9 (0.3, 7.9)	2.5 (0.4, 7.5)	0.908
Sputum lymphocytes, %	1.3 (0.5, 3.8)	2.1 (0.5, 5.7)	0.184

Data are presented as mean ± SD or median (IQR), unless otherwise indicated

*ACO* Asthma-COPD overlap, *NA* neutrophilic asthma, *EA* eosinophilic asthma, *MGA* mixed granulocytic asthma, *PGA* paucigranulocytic asthma, *IQR* interquartile range

### Associations of sputum eosinophil count with clinical characteristics

**Fig. 1:**
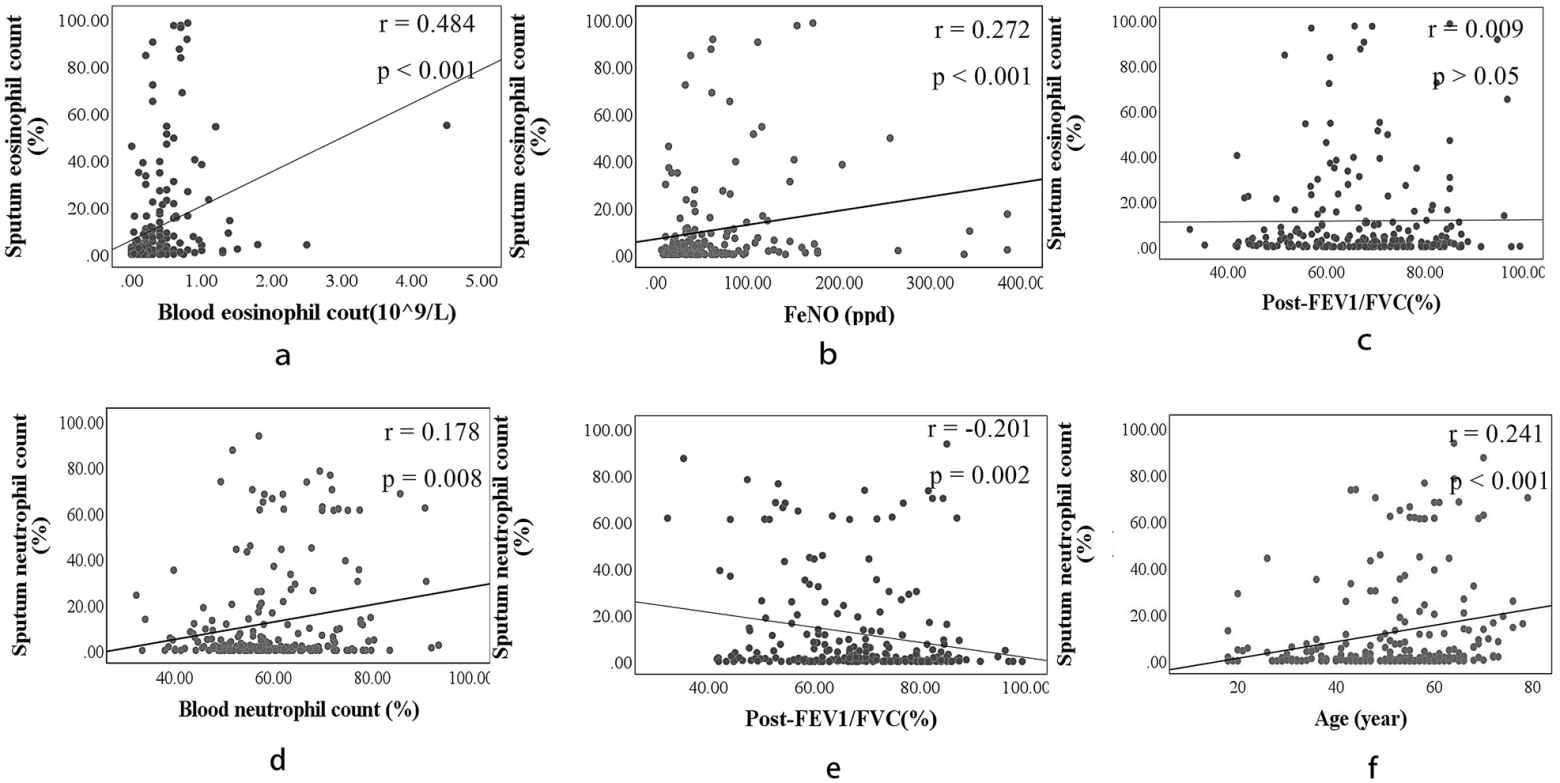
Spearman analysis of associations of sputum eosinophil/neutrophil count with clinical characteristics

**Fig. 2:**
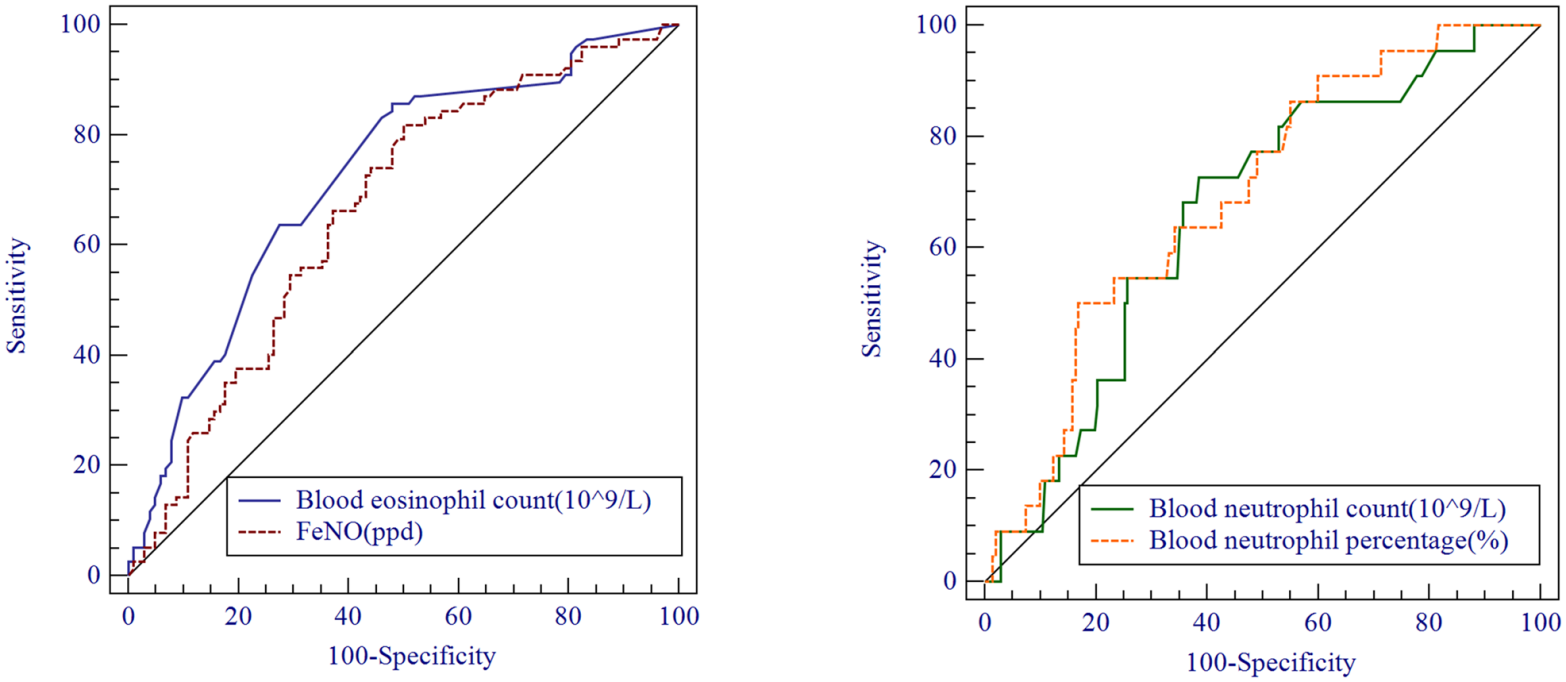
Receiver-operating characteristic (ROC) curve for all patients (n = 232) to determine the optimal cut-off values that best identified sputum eosinophilia ≥ 3% and sputum neutrophilia ≥ 61%. **a** Optimal cut-off values of blood eosinophil count and FeNO were 0.2 × 10^9^/L (sensitivity: 85%, specificity: 57%, AUC = 0.744, p < 0.001) and 30.25 ppd (sensitivity: 81%, specificity: 49%, AUC = 0.653, p < 0.001). **b** Optimal cut-off values of blood neutrophil count/proportion were 4.7 × 10^9^/L or 69.3% (sensitivity: 73%, specificity: 61%, AUC = 0.663, p = 0.012; sensitivity: 50%, specificity: 83%, AUC = 0.691, p = 0.003)

The ACQ6 score was positively correlated with the sputum eosinophil count (r = 0.187, p = 0.007), whereas the ACT score was negatively correlated with the sputum eosinophil count (Spearman’s r = 0.174, p = 0.011) (Fig. 1c). Importantly, blood eosinophil count and proportion were both significantly positively correlated with sputum eosinophil proportion (r = 0.484, p < 0.001; r = 0.451, p < 0.001) (Fig. 1a). ROC curve analysis showed that, to identify the presence of sputum eosinophil proportion ≥ 3%, the optimal cut-off values for blood eosinophil count were 0.2 × 10^9^/L or 2.5% (sensitivity: 85%, specificity: 57%, AUC = 0.744, p < 0.001; sensitivity: 81%, specificity: 59%, AUC = 0.733, p < 0.001) (Fig. 2a). In addition, the FeNO level showed positive correlation with sputum eosinophil proportion (r = 0.272, p < 0.001) (Fig. 1b). ROC curve analysis indicated that, to identify the presence of sputum eosinophil proportion ≥ 3%, the optimal cut-off value for FeNO was 30.25 ppd (sensitivity: 81%, specificity: 49%, AUC = 0.653, p < 0.001) (Fig. 2a). Notably, blood eosinophil count was positively correlated with FeNO level (r = 0.5, p < 0.001). Additionally, Z-test analysis using MedCalc statistical software revealed no significant difference in area under the curve (AUC) between FeNO and blood eosinophil count (p = 0.178).

### Associations of sputum neutrophil count with clinical characteristics

Analyses of all patients revealed negative correlations of sputum neutrophil count with AQLQ score, post-FEV_1_/FVC and post-FEV1 predicted value (r = − 0.145, p = 0.037; r = − 0.201, p = 0.002; r = − 0.165, p = 0.013) (Fig. 1e), but a positive correlation with age (r = 0.241, p < 0.001) (Fig. 1f). In addition, sputum neutrophil count was positively correlated with blood neutrophil proportion (r = 0.178, p = 0.008), but not with blood neutrophil count (r = 0.127, p = 0.059) (Fig. 1d). ROC curve analysis revealed that, to identify the presence of sputum neutrophil proportion ≥ 61%, the optimal cut-off values for blood neutrophil count were 4.7 × 10^9^/L or 69.3% (sensitivity: 73%, specificity: 61%, AUC = 0.663, p = 0.012; sensitivity: 50%, specificity: 83%, AUC = 0.691, p = 0.003) (Fig. 2b). In addition, blood neutrophil proportion was negatively correlated with the ACQ6 and FeNO (r = − 0.146, p = 0.032; r = − 0.157, p = 0.036).

### Logistic regression analysis to identify predictors of sputum eosinophilic/neutrophilic inflammation

Independent predictors of the presence of sputum eosinophilic inflammation were determined through multiple logistic regression models. When all variables were entered into the model, we found that blood eosinophil proportion and ACT score were identified as independent predictors of sputum eosinophil proportion ≥ 3% (log-transformed p < 0.001; p = 0.018) (Table 5). However, the FeNO level was not an independent predictor of sputum eosinophilic inflammation. Furthermore, blood neutrophil proportion and age were identified as independent predictors for sputum neutrophilic inflammation (sputum neutrophil proportion ≥ 61%) through regression analysis models (log-transformed p = 0.004; p = 0.006), while blood neutrophil count, FEV_1_/FVC and FEV_1_% predicted value were not (p > 0.05) (Table 5).

**Table 5:** Independent predictors of sputum eosinophilia and sputum neutrophilia/sputum neutrophilia

	Parameter	Β	SE	OR	95% CI	p value
Sputum eosinophilia	Blood eosinophil percentage, %	0.814	0.201	2.258	1.523–3.347	< 0.001
	ACT	− 0.426	0.178	0.653	0.463–0.930	0.018
Sputum neutrophilia	Blood neutrophil percentage, %	0.706	0.247	2.026	1.249–3.287	0.004
	Age	0.989	0.357	2.688	1.335–5.414	0.006

Sputum eosinophilia is defined as sputum eosinophil proportion ≥ 3%. Sputum neutrophilia is defined as sputum neutrophil proportion ≥ 61%

*SE* standard error

## Discussion

This study showed that patients with asthma from Jilin Province, China, had an older age at diagnosis of asthma, infrequent regular application of ICS, and worse lung function, relative to patients in other countries [18–21]. Furthermore, patients in this study exhibited worse asthma control and more patients with ACO [20, 22].

Our study showed that PGA was the most common inflammatory phenotype, followed by EA, MGA, and NA. Our results were similar to previous findings in Athens, Greece; in that study, the proportions of the four asthma phenotypes were paucigranulocytic (47.9%), eosinophilic (40%), mixed granulocytic (6.7%), and neutrophilic (5.4%) [23]. In contrast, a previous study in Australia revealed that EA, PGA, and NA were present in 41%, 32%, and 28% of the participants, respectively [17]. Furthermore, a study in Beijing, China, showed that neutrophilic (34.9%) and eosinophilic (34.9%) were the most frequent asthma phenotypes among all participants, while PGA (6.3%) represented the lowest proportion [24]; this finding differed from our results. The age, ICS treatment, and smoking history of patients with asthma may have been important influences on the asthma inflammatory phenotype distribution in this study.

In our study, ICS treatment and age were correlated with sputum neutrophilia among patients with asthma. Notably, age was positively correlated with sputum neutrophilia; patients with NA were significantly older than those with EA or PGA. Our findings are similar to those previously reported by Thomas et al. and Brooks et al. These prior studies revealed that age was significantly associated with sputum neutrophilia in adults, and that older patients more frequently exhibited NA [25, 26]. Furthermore, smoking has been shown to increase neutrophilic inflammation in sputum [27]. Our study showed that the proportion of patients who smoked was highest among patients with NA. In addition, patients with NA used a larger dose of ICS for a longer period of time in the past year; the dose of ICS treatment increased with the proportion of patients who exhibited NA. Cowan et al. previously reported that ICS treatment could increase the sputum neutrophil count in patients with asthma [28], possibly through inhibition of neutrophil apoptosis [29]. Simpson et al. reported that macrophage phagocytosis was severely impaired in patients with non-eosinophilic asthma [30]; we also observed that sputum macrophages were significantly reduced in patients with NA, which may explain the persistent elevation of airway neutrophil counts in these patients. Importantly, a greater proportion of patients had ACO (50.8%) in this study. Our study showed that patients with ACO were older and had a significantly higher proportion of smokers, as well as a greater smoking pack-year value. Patients with ACO had been treated with higher doses of ICS in the past year; they also had worse lung function and asthma control. In addition, patients with ACO exhibited more neutrophilic inflammation in sputum. Previous studies found that patients with smoking tobacco had higher odds of having ACO [31]. In vitro, simultaneous exposure of cigarette smoke extract and ovalbumin resulted in specific DNA methylation changes of MPV17L/ZNF323 genes, while may constitute a determinant for ACO [32]. Therefore, the higher proportion of smokers, poor patient compliance, insufficient treatment, and poor asthma control may have been the main causes of gradual progression to COPD, which led to a greater proportion of patients with ACO in this study.

Consistent with the findings in previous reports [33], we found that AQLQ score, FEV_1_/FVC, and FEV_1_% predicted value exhibited negative correlations with sputum neutrophil counts, which confirmed that the sputum neutrophil count was associated with severe [34, 35] and refractory [36] disease in patients with asthma. In our study, 44.4% of patients with NA exhibited severe disease. Furthermore, blood neutrophil count/proportion exhibited weak positive correlations with sputum neutrophil count. Our study indicated that age was an independent risk factor for sputum neutrophilia (95% CI 1.335–5.414; p = 0.006). Interestingly, blood neutrophil proportion, instead of count, was an independent risk factor associated with sputum neutrophilia (p = 0.004 vs p = 0.078). In addition, our research found that blood neutrophil proportion was negatively correlated with the ACQ6 and FeNO (p = 0.032; p = 0.036). The optimal cut-off value was 69.3% (sensitivity: 50%, specificity: 83%, p = 0.003) for identification of sputum neutrophilia, but the sensitivity was poor. These findings differed from the results in previous literature, which indicated that blood neutrophil count was not an independent risk factor for management of asthmatic airway neutrophilic inflammation [37]. Whether the blood neutrophil count can be used to monitor airway inflammation in asthmatic patients still needs to be further explored. However, AQLQ score, FEV_1_/FVC, and FEV_1_% predicted value were not independent risk factors for sputum neutrophilia in our study.

Previous studies have shown that PGA is a mildly inflammatory asthma phenotype [21, 23]. In our study, patients with PGA exhibited the lowest FeNO levels and best asthma control. Although our data also revealed that patients with PGA exhibit better lung function and lower sputum total cell count, our findings were more similar to those of prior studies, compared with the results described by Ntontsi et al. [23]. PGA was the most frequent phenotype in our study. PGA may be related to a good response to ICS treatment [23]. There were 112 (48.3%) patients who had used ICS regularly in the past year, which may have led to a high proportion of patients with PGA in this study. A previous study showed that patients with PGA had a higher sputum eosinophil count than healthy individuals, implying low-grade eosinophilic inflammation in those patients [21]. Therefore, some patients with paucigranulocytic inflammation in this study may be EA without recent exposures, or EA that was well controlled with ICS treatment. In addition, the cut-off points for EA in this study, sputum eosinophil proportion ≥ 3%, was higher than the values in some prior studies [17, 38], which may have contributed to the high PGA proportion in this study.

Patients with EA exhibited the highest FeNO level and worse asthma control; additionally, these patients included a lower number of smokers. Among patients with EA, 45.9% exhibited severe disease. In addition, ICS treatment has been shown to successfully reduce eosinophilic airway inflammation in patients with asthma, consistent with the results of a prior study [39]; fewer patients with EA were present in the high-dose ICS treatment group. As previously shown [9, 21], patients with EA had the highest numbers of blood eosinophils. Notably, the blood basophil count was elevated in patients with EA, which revealed concomitant enhancements of circulating eosinophils and basophils in patients with allergic asthma [40].

Our results showed that the sputum eosinophil count was negatively correlated with ACT score and lung function. Importantly, blood eosinophil count and FeNO level were positively correlated with sputum eosinophil count. Sputum eosinophil count also showed a stronger correlation with blood eosinophil count, compared with FeNO level; however, both correlations were not strong. The blood eosinophil thresholds demonstrated effectiveness similar to that of FeNO level for prediction of sputum eosinophilia. Our blood eosinophil thresholds were similar to the results reported by Schleich et al. (220/mm^3^ and 3%) [37] and Zhang et al. (0.26 × 10^9^/L and 2.7%) [9], but the FeNO level differed from the results reported by Schleich et al. (41 ppd) [37] and Alvarez-Puebla et al. (21 ppd) [41]. Therefore, the use of blood eosinophil count to identify sputum eosinophilia may be more stable than the use of FeNO level. Yap et al. reported that blood eosinophil count could be effectively used to predict sputum eosinophilia, whereas FeNO level could not [19]. Moreover, Hilvering et al. [8] and Alvarez-Puebla et al. [41] found that the predicted value of FeNO was more suitable for patients with asthma who were not receiving treatment with ICS. However, both blood eosinophil count and FeNO exhibited poor specificity in this study, which was consistent with the findings by Hastie et al. [42], although specificity was better in other studies [9, 37, 41]. Blood eosinophil proportion was an independent risk factor for sputum eosinophilia in our study, whereas FeNO level was not.

The inflammatory phenotype of patients with asthma is reportedly influenced by treatment, especially involving ICS [43]. An important limitation of our study was that all patients in this study underwent sputum induction immediately after enrollment to reduce the potential effect of treatment on the asthma inflammatory phenotype, but this did not completely rule out the effect of treatment among those patients.

## Conclusions

The most common asthma phenotype in this study was PGA, followed by EA. Higher proportion of smokers, poor patient compliance, insufficient treatment, and poor asthma control may have been the main causes of high ACO proportion among patients in this study. Blood eosinophil/neutrophil counts exhibited poor specificity and sensitivity for prediction of airway eosinophilic/neutrophilic inflammation. Further studies are needed to confirm whether full blood cell counts can achieve sufficient accuracy to predict asthma inflammatory phenotype.

## Data Availability

All data generated or analyzed during this study are included in this article.

## Abbreviations

GINA: Global Initiative for Asthma
FeNO: Fractional exhaled nitric oxide
ACQ6: 6-Item Asthma Control Questionnaire
ACT: Asthma Control Test Questionnaire
AQLQ: Asthma Quality of Life Questionnaire
HADS: Hospital Anxiety and Depression scale
ROC: Receiver operating characteristic
AUC: Area under the curve
FVC: Forced vital capacity
PGA: Paucigranulocytic asthma
EA: Eosinophilic asthma
MGA: Mixed granulocytic asthma
NA: Neutrophilic asthma
TCC: Sputum total cell count
